# What does matrix metalloproteinase-1 expression in patients with breast cancer really tell us?

**DOI:** 10.1186/1741-7015-9-95

**Published:** 2011-08-11

**Authors:** Ferdinando Mannello

**Affiliations:** 1 Department of Biomolecular Sciences, Section of Clinical Biochemistry, Unit of Cell Biology, University 'Carlo Bo' of Urbino, I-61029 Urbino (PU), Italy

## Abstract

Molecular and biochemical expressions of matrix metalloproteinases in breast cancer tissue and cells offers promise in helping us understand the breast cancer microenvironment, and also in the future it is hoped this will improve its detection, treatment and prognosis. In a retrospective study recently published in *BMC Cancer*, microenvironment predisposing to breast cancer progression, metastatic behavior and the expression of matrix metalloproteinase-1 (MMP-1) and its correlation with well-known biochemical, molecular and clinicopathologic factors in breast cancer cells and cancer-associated stromal cells was examined; this study also analyzed patient survival in different breast cancer subtypes. The positive correlation in breast tumor and stromal cells between MMP-1 expression and several markers of tumor grade and stage provide us with some useful new insights into important questions about the molecular profiling of the stromal microenvironment in metastatic breast cancer. The study showed that MMP-1 expression is strongly associated with poor clinical outcome, so now we look forward to future larger studies in breast cancer patients in which we can relate wider MMP molecular profiling to identify lethal tumor and stromal microenvironments predisposing to breast cancer progression, metastatic behavior and poor prognosis.

Please see related article http://www.biomedcentral.com/1471-2407/11/348

## Introduction

Breast cancer (BC) is the most common cancer in women worldwide, comprising at least 16% of all female cancers. BC results from multiple environmental and hereditary risk factors, even though genetic traits, age and hormones are the main recognized BC-predisposing risk factors [1]. Human female BC encompasses a variety of tumors, which differ in their morphological, biochemical and molecular characteristics, all guiding clinical outcome and patient survival. Although well-documented classic diagnostic/prognostic biomarkers/profiles are reliable (for example, tumor grade and stage, p53, bcl-2, Ki-67, hormone receptor status,: human epidermal growth factor receptor 2 (HER-2) expression), there is the urgent need to differentiate between BC subclasses (for example, non-basal-like luminal A and B, basal-like, triple-negative BC)[2,3], patients with different prognoses and treatment responses to the same therapy [4-6].

**Figure 1:**
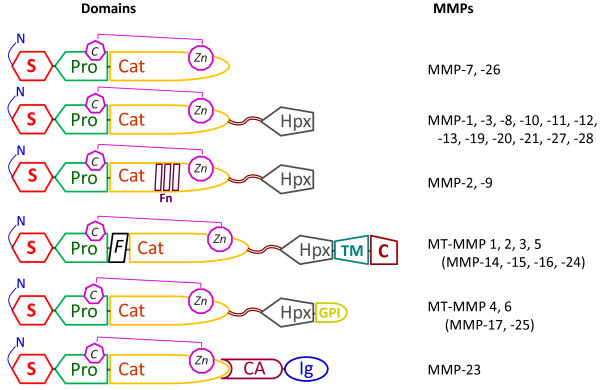
**Domain structures of secreted and membrane-anchored MMPs**. The basic organizations of human MMP family members are depicted: S, signal peptide; Pro, pro-peptide; Cat, catalytic domain, containing cysteine group (C);Zn, zinc ion; Fn, fibronectin-II- like repeats; Hpx, hemopexin like domain; TM, transmembrane domain; GPI, glycol-phosphatidylinositol membrane anchor; C, cytoplasm tail; CA, cysteine array; Ig, immunoglobulin-like domain; the flexible linker or hinge region is represented by a wavy black ribbon. The domain structure includes the signal peptide, which guides the enzyme into the endoplasmic reticulum during synthesis, the propeptide domain, which sustains the latency of MMPs, the catalytic domain, which houses the Zn2+ region and is responsible for enzyme activity, the hemopexin domain, which determines the substrate specificity, and a small hinge region. Additional transmembrane and intracellular domains are also present: the hinge region in MMP-9 is heavily O-glycosylated; the furin-activated MMPs and all of the membrane-anchored MMPs have a basic motif at the C-terminal end of their prodomains;; the two gelatinases (MMP-2 and -9) contain three fibronectin-II-like repeats; four of the six MT-MMPs are anchored to the cell membranes through a type I transmembrane domain and the other two through a glycosylphosphatidylinositol moiety. The membrane-anchored MMP-23, has an N-terminal type II transmembrane domain. The two minimal domain MMPs and MMP-23 lack the HPX domain and, in the latter enzyme, this domain is replaced by a C-terminal cystein array (Ca) and an immunoglobulin-like (Ig) domain. MMPs are produced in a latent form and most are activated by extracellular proteolytic cleavage of the propeptide and finely regulated by the tissue inhibitors of metalloproteinases (TIMP)[9].

Examining new BC biomarkers has proven that matrix metalloproteinases (MMP), which are zinc-dependent endopeptidases belonging to the *Metzincin *superfamily, are involved in several key events of both physiologic processes (for example, tissue remodelling, stem cell differentiation and proliferation, apoptosis) [7-11] and in pathological conditions (for example, inflammation, degeneration and cancer) [12-14]. The MMP family comprises several classes of proteases [15], which cleave almost all extracellular matrix components and a variety of proteins and growth factors crucial for neoplastic initiation and progression; these data suggest MMPs as good targets for tumor biomarker discovery. In humans, there are 24 MMP genes, but only 23 MMP proteins [16], including 17 soluble, secreted enzymes and 6 membrane-associated proteinases. MMPs are built up by a diverse structural domain architecture, and differ in their substrate specificity and in temporal and tissue specific expression patterns. MMPs were originally named for their preferred substrates within the extracellular matrix (ECM): collagen-cleaving MMPs (MMP-1, -8, and -13) were designated collagenases, gelatin (denatured collagen)-cleaving MMPs (MMP-2 and -9) were termed gelatinases, and MMPs degrading a broad spectrum of ECM proteins were called stromelysins (MMP-3, -10, and -11) or matrilysins (MMP-7). As the MMP family grew with the discovery of additional paralogs, including the membrane-associated MMPs, a numbering system was adopted, and MMPs are now grouped according to their domain structure (Figure 1).

Many studies have characterized the increased expression of MMPs at both protein and mRNA levels, identifying them as a key event leading to the initiation/progression of BC and linking them with the ability of cancer to metastasize. Thus, MMPs could be used as tumor biomarkers and indicators of cancer metastasis with diagnostic and prognostic usefulness [17,18]. On the other hand, recent evidence underlines the fact that some MMPs (such as MMP-8, also named collagenase-2 or neutrophil collagenase) may favor host defense instead of stimulating tumor proliferation, suggesting that these proteinases have an unexpected protective biological role in cancer processes [19,20].

Among the MMP members involved in BC, both the biochemical and molecular expression profile of MMP-1 (named also collagenase-1 or interstitial collagenase) have been extensively analyzed in human BC. Although there are a great number of valuable *in vitro *and *in vivo *studies concerning its role in breast carcinogenesis (see reviews [21-23]), some parts of its regulation and expression (as an assisting marker in metastatic BC diagnosis) remain poorly understood and a matter of debate [21,24,25].

To address this hot topic, a recent study published in *BMC Cancer *[26] has retrospectively examined MMP-1 expression in both breast cancer cells and cancer-associated stromal cells from BC patient to evaluate the relationship among MMP-1 and classic prognostic factors, analyzing the extensively long follow-up time for cancer specific survival in different BC subtypes.

## Discussion

**Figure 2:**
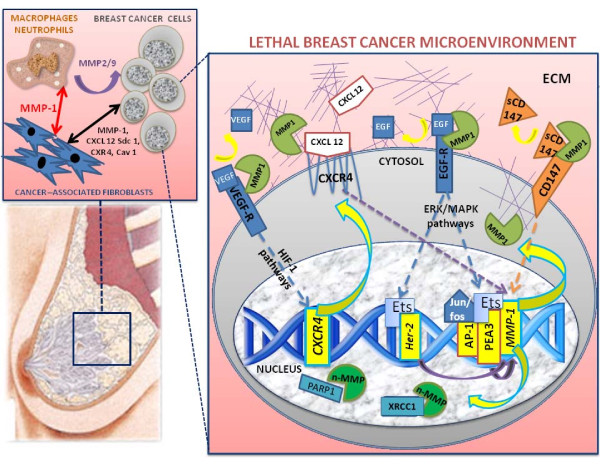
**Schematic view of the role and functions of MMP-1 in breast cancer microenvironment**. The breast microenvironment is composed of ductal and luminal epithelial cells sitting on a layer of myoepithelial cells, and stromal cells (including leukocytes, fibroblasts, and endothelial cells). The microenvironment alterations during breast cancer (BC) progression lead to a decrease of myoepithelial cells, phenotypically altered because of signals coming from the tumor and stromal cells. The cell-cell and cell-matrix interactions during BC initiation/progression involve the interplay among cancer-associated fibroblasts, macrophages and BC cells. The secretion of MMP-1 and the release of soluble proteins (including EGF, VEGF, CD147, syndecan-1) in the breast milieuare able to activate tumor pathways, generating the 'lethal BC microenvironment', which triggers a 'vicious cycle' between tumor and stromal cells, enhancing the BC growth, and promoting the invasive and metastatic processes via paracrine interactions [13]. The unexpected roles of MMP-1 interstitial collagenase are related to the proteolytic activity in the ECM compartment and the cell-cell interactions. The unexpected nuclear localization of some MMPs has been recently linked to new functional roles of these proteases within nuclei of tumor cells, with the ability to cleave peculiar nuclear peptides (Mannello et al, ms in preparation). The proteolytic activity of MMP-1 in BC cells is linked to the cleavage/release of soluble VEGF, EGF, CXCL 12 and CD147 from their receptor complexes. These proteins are able to activate crucial cancer metabolic routes (like HIF-1-dependent and MAPK and ERK-dependent pathways) that promote BC proliferation, triggering a vicious cycle through the molecular activation of some transcription factors (like AP-1, PEA3 and Ets), indispensable switches for the HER-2, CXCR4 and MMP-1 gene regulation [39]. The secretion of MMP-1 is also able to activate other MMPs (like MMP-2 and -9) which have been widely recognized as crucial steps for the BC evolution [14].

The highly complex BC tissue is composed of neoplastic cells and stromal cell compartments [27], containing a variety of mesenchymal cells (notably fibroblasts, myofibroblasts, endothelial cells and inflammatory cells associated with the immune system) (Figure 2). The specific contributions of the cancer-associated fibroblasts to tumor growth are poorly understood, but it has been suggested that they are able to promote the growth of mammary carcinoma cells and to enhance tumor angiogenesis [28-30], as well as through the secretion of proteinases (including MMP-1) and other proteins (for example, stromal-cell derived factor 1 (also called CXCL 12), syndecan-1, CXCR4 and Caveolin-1) [31,32]. The molecular profile of the lethal breast cancer microenvironment is based on activated cancer-associated fibroblasts and revealed also by high levels of interstitial collagenase MMP-1 (both at protein and mRNA levels), which facilitate angiogenesis and increase ECM degradation, both crucial processes for the invasive and migratory phenotype of metastatic BC [14]. Interestingly, it has also been demonstrated that human epidermal growth factor receptor 2 (HER-2) induces MMP-1 expression through the enhanced regulation of the transcription factor/proto-oncoprotein Ets-1, suggesting that (contrary to the protective role of MMP-8 neutrophil collagenase [20]) the interstitial collagenase MMP-1 may significantly affect the metastatic behavior of BC cells [33]. The same biochemical behaviors and biological functions of both MMP-1 and -8 collagenases have also been detected in plasma samples collected from BC patients with poor prognosis [34].

The recent study of Boström and Colleagues [26] is helpful in that it sought to link the expression of MMP-1 with a broader picture of immunohistochemical biomarker changes, and their relationships between BC and stromal cells. According to the well-established role of MMP-1 in BC promotion and outcome [21,35], the authors claim that the independent prognostic value of MMP-1 is mainly based on the positive correlations with tumor grade and p53 positivity in tumor and stromal cells; the peculiar expression of MMP-1 in stromal cells also showed a significant association of BC with HER-2 over-expression and triple negative BC. Furthermore, in the luminal B BC subtype (estrogen receptor and/or progesterone receptor positive, other than HER-2 positive), MMP-1 expression in stromal cells was higher than in the luminal A subtype (estrogen receptor and/or progesterone receptor positive, and HER-2 negative). Also, the luminal B subtype stromal cells showed higher MMP-1 expression when compared to triple negative BC cells (identified as a basal-like subtype in about 70% of cases). From the more than 20 years survival analyses, Boström and colleagues revealed that there were statistically significant differences in BC-specific survival among women with tumors with high versus low expression of MMP-1, tumor grade I versus III, triple-negative versus non-triple negative, basal-like versus non-basal-like tumors, and low versus high Bcl-2 and Ki-67 expressions. All these data reveal that high MMP-1 positivity in both stromal and tumor cells was significantly associated with tumor evolution, poor prognosis and shortened survival. Noteworthy also is the higher MMP-1 expression in both the cytoplasm and nuclei of BC cells with respect to stromal cells, adding new information to the poorly characterized and unexpected role of nuclear MMPs (Mannello et al, unpublished observations), suggesting them not just for ECM anymore [11]. In this respect, it has recently been suggested that some MMPs (such as the MMP-3 stromelysin and MMP-2 gelatinase) may be involved in transcriptional gene regulation [36] and the cleavage of poly-ADP-ribose-polymerase [37].

Recent studies highlight the biological importance of stroma in breast physiopathology [38], suggesting that the gene expression and regulation of some MMPs are largely restricted to the stromal compartment [39]. Although the role of the stromal compartment in BC had been originally depicted as the Cinderella of the cancer biology, even more evidence has supported the crucial role of stromal cells in tumor evolution and then in BC patient survival. Early studies showed that the normal mammary microenvironment is capable of reverting the malignant phenotype of BC cells by inducing a more differentiated state, suggesting that cancer cells may only thrive in an abnormal environment in which they evolved [40]. Pathologists have also long noted the prognostic value of certain histopathological features of BC (including lymphocytic infiltration and angiogenesis), suggesting a role for non-epithelial cells in carcinogenesis, as shown by differences in tumor initiation and progression depending on the variability in germline genotypes and phenotypes [41].

Despite the convincing observations implicating a role for microenvironmental and systemic alterations in breast tumorigenesis (reviewed in [42]), our understanding of the genes and metabolic pathways mediating cellular interactions and paracrine regulatory networks among various cell types in both normal and neoplastic breast tissue is still limited. However, it has been clearly demonstrated that gene expression changes occur in all cell types during breast tumor progression, but clonally selected genetic alterations are restricted to tumor epithelial cells [43]. Interestingly, the comparison of myoepithelial cells from normal breast tissue with ductal carcinoma *in situ *(DCIS) yielded the highest number of consistently differentially expressed genes; a significant fraction of these encoded for secreted proteins and cell surface receptors, suggesting intensive autocrine/paracrine regulatory loops in the breast pre-cancer and cancer microenvironment [27,30,31,42].

It has recently been demonstrated that the gene expression signature of epithelial cells correlated with tumor grade but not with histologic stage, whereas genes up-regulated in tumor-associated stroma included many ECM-related molecules (including MMP-1 collagenase), expressed at higher levels in invasive compared with *in situ *tumors [44,45]. In particular, it has been recognized that, among the mesenchymal cells constituting the stroma, both the cancer-associated fibroblasts and the inflammatory cells associated with the immune system are able to drive cancer initiation and progression through peculiar biochemical pathways and molecular signatures, significantly different between *in situ *and invasive BC [42,46]. The dramatic changes in gene expression patterns, but the lack of clonally selected somatic genetic alterations in the tumor-associated myoepithelial and stromal cells, has also suggested potential epigenetic alterations; this may be because stromal cells isolated from normal and tumor tissues are known to maintain their differences even after prolonged cell culture and in xenograft studies (as reviewed in [38]).

The historically prevailing view of BC progression is focused on tumor epithelial cells, whereby gradual progression of a tumor through defined steps is entirely due to the accumulation of both genetic and epigenetic alterations that confer progressively malignant phenotypes [47]. However, this model has been challenged after multiple studies demonstrating the importance of the microenvironment in shaping tumor evolution and progression through the new functions of several MMPs (including MMP-1 collagenase)[42,47-50].

With this background, the molecular and biochemical profiling of a lethal BC microenvironment has led to the identification of new functions of MMP-1 in the stromal microenvironment of BC, revealing a significant link between cancer-associated fibroblasts and MMP-1 expression with metastatic tumor progression and/or poor clinical outcome [21]. In particular, the paper of Boström and colleagues [26] sheds further light on these relationships, demonstrating significant differences of MMP-1 expression by cancer-associated stromal cells in luminal A, luminal B and triple-negative BC subclasses. The importance of MMP-1 expression and its cellular localization (other than the unexpected presence of nuclear MMP-1) in BC stromal cells is in agreement with the functional and clinical relevance of microenvironmental alterations in breast tumorigenesis. Their results support the molecular evidence that high MMP-1 mRNA expression and both aplotypes and polymorphisms of MMP-1 promoter gene may represent a risk factor in patients with invasive BC [51], and recognize MMP-1 as a prognostic marker in patients with invasive/metastatic BC [52-54]. Thus, Boström's study provides stronger evidence that the expression of MMP-1 in stromal fibroblasts of BC help to identify patients with atypical tumor-stromal fibroblasts in which MMP-1 may contribute to BC invasiveness and metastatic behavior.

## Conclusions

Boström and colleagues provide us with a stronger scientific basis for understanding the involvement and cooperation of both tumor and stromal cells in BC progression and outcome, as well as the role of MMP-1 expression (both in cytoplasm and nuclei) for metastatic dissemination, identifying BC women with shortened relapse-free survival and poor outcome.

Although we are now learning that the useful relationship among MMP-1 expression and well-known clinic/pathologic-prognostic factors (such as Ki-67, HER-2, Bcl-2, tumor grade and cancer subtypes) may help us to enhance and increase the clinically useful prognostic factors, what we need are future biochemical, molecular and clinical studies assessing many different biomarkers head-to-head, because MMP-1 analysis alone is no longer the way to go.

In this respect, the perspectives should involve studies focused on gene and protein expression profiling of stromal alterations associated with BC progression, identifying key transcriptional changes that occur early in cancer initiation/development. For example, a possible synopsis for future research could include:

1. The profile of the MMP superfamily (including the proteomic analysis of both protective and dangerous MMPs). This research will evaluate the kind of MMP which is involved in the proteolytic cascade cleaving/regulating not only structural components of both ECM and nuclear compartments, but also the modulation of several growth factor precursors, cell surface receptors, cytokines and cell adhesion molecules; on the other hand, we should obtain information about the MMPs involved in tumor-suppression [20].

2. The analysis of tissue inhibitors of metalloproteinase (TIMPs) (well-known inhibitors of MMPs also characterized by MMP-independent functions in cancer biology [9,55]). This approach allows the analysis of the proteolytic/antiproteolytic balance, as a cancer/metastatic specific switch). It will be interesting for future studies to identify processes that control the earlier stages of disease progression, helping to decipher the molecular and biochemical mechanisms underlying cellular and microenvironmental interactions in the breast tumors.

3. The screening of both MMP gene aplotypes and polymorphisms, to obtain a phamacogenomic profile of the individual variation in drug response and therapeutic efficacy [56]. Considering the potential contribution of BC stromal microenvironmental alterations on MMP-1 biochemical and molecular alterations, the knowledge of MMP gene aplotypes and/or polymorphisms may help in the design of more efficient target therapy, limiting drug-resistance and underlining the importance of anti-target identification in drug development for blocking metastases [14].

4. The histochemical and molecular analysis of the nuclear localization of MMP-1 protein (in latent and/or active form) to evaluate its role during cancer associated apoptosis and possible therapeutic potential (Mannello et al, unpublished observations and manuscript in preparation).

As highlighted by Boström and colleagues, MMP-1 expression in both stromal and tumor cells may control BC progression, suggesting that BC metastasis and outcome are driven by complex and reciprocal interactions between epithelial cancer cells and their stromal microenvironment. The biochemical and molecular profiling of invasive BC will be crucial to identify a 'lethal tumor microenvironment' [57] associated with metastatic tumor progression and/or poor clinical outcome for the leading cause of cancer death among women in high-income countries [1]. It is noteworthy that the 'lethal tumor microenvironment' is likely to have an impact on numerous solid tumors in different parts of the body, widening the impact of the results of Boström and colleagues to other fields of oncology (such as in breast, lung, pancreas and haematological malignancies)[50,58]. In fact, the tumor microenvironment alterations in human cancer not only influence tumor progression and predict prognosis, but also have major effects on the efficacy of cancer therapy, especially the targeted therapy aimed at growth factor receptors and secreted proteins, such as HER-2 and MMP-1. Modifications in the cancer microenvironment may alter the fitness landscape providing a possible growth advantage for cells with tumor-initiating genetic-epigenetic changes [59,60].

Ultimately, the study of Boström and Colleagues opens new possibilities and the ability to specifically target the expression of MMP-1, a particular MMP involved in BC initiation/progression, aberrantly expressed in the metastatic process, suggesting further diagnostic, prognostic and therapeutic potential.

## Abbreviations

BC: breast cancer; DCIS: ductal carcinoma in situ; HER-2: human epidermal growth factor receptor 2; MMP: matrix metalloproteinase; TIMP: tissue inhibitors of metalloproteinase.

## Competing interests

The author declares that they have no competing interests.

## Pre-publication history

The pre-publication history for this paper can be accessed here:

http://www.biomedcentral.com/1741-7015/9/95/prepub
